# A multicenter prospective cohort study of cardiac ultrasound phenotypes in patients with sepsis: Study protocol for a multicenter prospective cohort trial

**DOI:** 10.3389/fmed.2022.938536

**Published:** 2022-07-27

**Authors:** Hongxuan Zhang, Xiaoting Wang, Wanhong Yin, Hongmin Zhang, Lixia Liu, Pan Pan, Ying Zhu, Wei Huang, Zhiqun Xing, Bo Yao, Cui Wang, Tianlai Lin, Rongguo Yu, Xiuling Shang

**Affiliations:** ^1^The Third Department of Critical Care Medicine, Shengli Clinical Medical College of Fujian Medical University, Fujian Provincial Hospital, Fujian Provincial Center for Critical Care Medicine, Fujian Provincial Key Laboratory of Critical Care Medicine, Fuzhou, China; ^2^Department of Critical Care Medicine, Peking Union Medical College, Chinese Academy of Medical Sciences, Beijing, China; ^3^Department of Critical Care Medicine, West China Hospital, Sichuan University, Chengdu, China; ^4^Department of Critical Care Medicine, The Fourth Hospital of Hebei Medical University, Shijiazhuang, China; ^5^Department of Respiratory and Critical Care Medicine, Chinese PLA General Hospital, Beijing, China; ^6^Department of Critical Care Medicine, Affiliated Hangzhou First People's Hospital, Zhejiang University School of Medicine, Hangzhou, China; ^7^Department of Critical Care Medicine, School of Medicine, First Affiliated Hospital of Xiamen University, Xiamen University, Xiamen, China; ^8^Department of Critical Care Medicine, Shandong Provincial Hospital Affiliated to Shandong First Medical University, Jinan, China; ^9^Department of Critical Care Medicine, The Affiliated Hospital of Qingdao University, Qingdao, China; ^10^Department of Critical Care Medicine, The First Affiliated Hospital of Anhui Medical University, Hefei, China; ^11^Department of Critical Care Medicine, Quanzhou First Hospital Affiliated to Fujian Medical University, Quanzhou, China

**Keywords:** sepsis, septic cardiomyopathy, ultrasound, cardiac phenotype, prognosis

## Abstract

**Background:**

Sepsis-induced cardiomyopathy significantly increased the mortality of patients with sepsis. The diagnostic criteria for septic cardiomyopathy has not been unified, which brings serious difficulties to clinical treatment. This study aimed to provide evidence for the early identification and intervention in patients with sepsis by clarifying the relationship between the ultrasound phenotype of septic cardiomyopathy and the prognosis of patients with sepsis.

**Methods:**

This was a multicenter, prospective cohort study. The study population will consist of all eligible consecutive patients with sepsis or septic shock who meet the Sepsis 3.0 diagnostic criteria and were aged ≥18 years. Clinical data and echocardiographic measurements will be recorded within 2 h, at the 24th hour, at the 72nd hour, and on the 7th day after admission. The prevalence of each phenotype will be described as well, and their association with prognosis will be analyzed statistically.

**Discussion:**

To achieve early recognition, prevent reinjury, achieve precise treatment, and reduce mortality in patients with sepsis, it is important to identify septic cardiac alterations and classify the phenotypes at all stages of sepsis. First, there is a lack of studies on the prevalence of each phenotype in Chinese populations. Second, each phenotype and its corresponding prognosis are not clear. In addition, the prognosis of patients with normal cardiac ultrasound phenotypes vs. those with suppressed or hyperdynamic cardiac phenotypes is unclear. Finally, this study was designed to collect data at four specific timing, then the timing of occurrence, duration, changes over time, impact to outcomes of each phenotype will probably be found. This study is expected to establish a standard and objective method to assess the ultrasound phenotype of septic cardiomyopathy due to its advantages of visualization, non-invasiveness and reproducibility, and to provide more precise information for the hemodynamic management of septic patients. In addition, this research will promote the clinical application of critical care ultrasound, which will play an important role in medical education and make ultrasound the best method to assess cardiac changes in sepsis.

**Trial registration:**

https://clinicaltrials.gov/ct2/show/NCT05161104, identifier NCT05161104.

## Introduction

Sepsis is a life-threatening organ dysfunction resulting from a dysregulated host response to infection (1). The heart is one of the most important organs for oxygen supply and consumption and is frequently involved in sepsis. Septic myocardial suppression increases mortality in patients (2). Recent studies have found that a hyperdynamic state of the left ventricle (left ventricular ejection fraction [LVEF] >70%) is associated with mortality in intensive care unit (ICU) patients with sepsis, possibly because it reflects unresolved vascular paralysis from sepsis (2). For septic myocardial suppression, there is still a lack of uniform criteria for diagnosis; however, it is well established that the cardiac ultrasound phenotype of septic myocardial suppression can be left ventricular systolic dysfunction (LVSD), left ventricular diastolic dysfunction (LVDD), right ventricular dysfunction (RVD), diffuse ventricular dysfunction, and mixed ventricular dysfunction. According to available literature statistics, the prevalence of LVSD ranges from 12 to 60%, the prevalence of LVDD is higher at 20% to 79%, and the prevalence of RVD varies from 30 to 55% (3). However, based on the current understanding of septic myocardial suppression, the relationship between each stage and its prognosis is unclear. Echocardiography can rapidly identify septic myocardial suppression and guide its classification, thus further optimizing the diagnosis and treatment process of sepsis, particularly to avoid over-resuscitation during fluid resuscitation and perform reverse resuscitation in a timely manner to improve patient prognosis and reduce hospitalization time. This study aimed to classify and evaluate the prognosis of patients with different septic cardiac ultrasound phenotypes in multiple centers across China by measuring the right and left heart systolic and diastolic indices through echocardiography, recording the baseline conditions and clinical indices of patients, and combining them with their prognosis. Thus, in this study, a standardized evaluation system was established to evaluate the cardiac ultrasound phenotype of sepsis patients for the early identification of septic cardiac changes and classification of cardiac phenotypes in various stages of sepsis and to further alleviate sepsis treatment problems caused by the disease. This study also demonstrates the benefits of ultrasound in clinical dynamics and individualized assessment, with medical students gaining not only a convenient tool in their education, but also the clinical thinking skills needed to match it.

## Methods

### Inclusion criteria

The present study will be conducted in several medical centers in China, including Fujian Provincial Hospital, West China Hospital of Sichuan University, Peking Union Medical College Hospital, Fourth Hospital of Hebei Medical University, Chinese PLA General Hospital, First Peopl*e*'s Hospital of Hangzhou City Affiliated to Zhejiang University, First Affiliated Hospital of Xiamen University, Shandong Provincial Hospital Affiliated to Shandong First Medical University, the Affiliated Hospital of Qingdao University, Quanzhou First Hospital affiliated to Fujian Medical University, the First Affiliated Hospital of Anhui Medical University, between April 2022 to April 2025. Informed consent will be obtained from all patients or their legal guardians. All patients with sepsis or septic shock who diagnosed using the Sepsis 3.0 diagnostic criteria (1) and were ≥18 years of age who admitted or hospitalized to each center will be included. Sepsis is defined as an increasing of ≥2 points from baseline in the Sequential Organ Failure Assessment (SOFA) score due to infection, and septic shock is defined as persistent hypotension on top of sepsis, requiring vasoactive drugs to maintain a mean arterial pressure ≥65 mmHg and a blood lactate level >2 mmol/L despite adequate fluid resuscitation. Patients will be admitted to the ICU, and transthoracic echocardiograms will be performed by relevant qualified personnel and interpreted by two qualified personnel. Two senior sonographers (A and B) will independently examine the patients and compare them to identify the true positives as accurately as possible. The agreement rate between the two specialists is as high as 100%, and the reconfirmation of the individual set with doubts reach a unified conclusion. In case of diagnostic disagreement, we will ask an expert panel for a resolution, and as a quality control, all ultrasound images and measurements of each enrolled patient will be recorded. In addition, the results will be compared with the latest guidelines before building the dataset. Patients eligible for inclusion will have their first echocardiogram examination completed within 2 h of admission. If eligible, informed consent will be required prior to formal study entry.

### Exclusion criteria

The exclusion criteria included patients with the following situations:

- Preexisting chronic heart disease, such as cardiomyopathy, chronic pulmonary heart disease, severe cardiac valve disease, coronary heart disease, congenital heart disease, and pericardial disease, and with cardiac function of grade ≥III (NYHA classification) prior to sepsis
- End-stage malignancies
- Severe trauma
- Pregnancy
- Patients for whom transthoracic echocardiography data are not available.

### Data acquisition

This study has been approved by the local ethics committee of every participated center. The continuous inpatient electronic medical records will be reviewed for sepsis between April 2022 to April 2025. The prevalence of septic myocardial suppression has been reported in previous literature as 10–70% (4), taking the middle value of 40%. With a 95% confidence level, the results were required to fall within 10% of the overall truth rate, and the required sample size was estimated to be at least 1,152 cases. Detailed demographic and clinical characteristics will be collected and recorded, including age, sex, height, weight, primary diagnosis, site of infection, etiology (if known), underlying disease (hypertension, diabetes, coronary artery disease, chronic kidney disease, chronic obstructive pulmonary disease, oncologic disease, autoimmune disease, etc.), and medication history (beta-blockers, digitalis analogs, hormones, immunosuppressive agents, antineoplastic drugs, etc.).

Clinical and laboratory data will be collected included temperature, heart rate, respiratory rate, peripheral oxygen saturation, blood pressure (systolic/diastolic), pH, partial pressure of oxygen, inspired oxygen concentration, Glasgow Coma Scale score, white blood cell count, neutrophil count, lymphocyte count, hematocrit level, platelet count, calcitonin level, G-test scores, serum albumin level, total bilirubin level, serum creatinine level, troponin I level, NT-proBNP level, maximum dose of vasoactive drugs (norepinephrine, dopamine, vasopressin) or cardiac drugs (dobutamine, levosimendan, desacetyl trichothecene), SOFA score, and APACHE II score.

The ultrasound parameters are as follows:

- Indicators of left ventricular systolic function: mitral annular plane systolic excursion (MAPSE), LVEF, and left ventricular outflow tract flow velocity time integral
- Indicators of left ventricular diastolic function: E, A, and *e*'
- Right ventricular function indicators: tricuspid annular plane systolic excursion (TAPSE), inferior vena cava end-expiratory and end-inspiratory internal diameters, and right/left heart diastolic basal segment transverse diameter ratio (apical four-chamber heart view)
- Basal status indicators: right ventricular free wall thickness (subxiphoid four-chamber cardiac section) and septal thickness (apical four-chamber cardiac section).

The hemodynamic parameters will be collected included the central venous pressure, central venous–arterial blood carbon dioxide partial pressure difference, central venous oxygen saturation, and arterial blood lactate concentration.

All parameters mentioned above will be recorded within 2 h after sepsis diagnosis in the ICU, followed by recording of the ultrasound parameters and hemodynamic parameters once at 24, 72 h, and 7 days.

The prevalence of each phenotype will be described firstly. The primary outcomes are in-hospital and 28-day mortality rates. The secondary outcomes are the degree of echocardiographic improvement on day 7, length of stay in the ICU, and number of days on mechanical ventilation. Severe and irreversible disease will be determined by the supervising physician, and the patient's family chose automatic discharge due to local customary conditions indicating death.

If a respondent is absent at a point in time when access is required, resulting in missing data at that point in time, or if an ending visit is missed, data from other points in time can be included in the analysis for that point in time without affecting the integrity and accuracy of the data at other points in time. We will ensure that the data collection was standardized and controllable, with the advantages of complete information, consistent structure, and no redundant information. In the process of data collection, we use the case report form to extract the clinical information required for the study from the research subjects, convert it into a standardized data form, and construct a reasonable database.

### Data statistical analysis

SPSS (version 23.0; SPSS Inc., Chicago, IL, USA) will be used for the analysis. Continuous variables will be defined as the mean ± standard deviation, and categorical variables will be presented as percentages. The Kolmogorov–Smirnov test will be used to verify the normality of the distribution of the continuous variables. One-way ANOVA will be used for comparison of means between groups; two independent samples *t*-test will be used for comparison between two groups; χ^2^ test will be used for counting data; and multifactor logistic regression and Cox survival analysis will be used to determine the factors of prognosis. P-values < 0.05 will be considered statistically significant.

The participants will be divided into a modeling cohort and a validation cohort in a 7:3 ratio. In the modeling cohort, variables with *P* < 0.05 in the univariate Cox analysis will be included in the multivariate Cox analysis, and nomogram plots will be constructed. Finally, the validation cohort will be used to evaluate the discrimination ability of the model based on the area under the ROC curve.

## Discussion

Several studies have confirmed the high mortality rate in patients with sepsis, ranging from 34 to 56% (5) and 33.5% (6) in foreign and domestic countries, respectively, and even higher in patients with combined hyperdynamic left ventricular function or myocardial depression (3, 7). Standardizing the staging of myocardial depression in sepsis and realizing the early identification of patients with sepsis are of great significance for clarifying the cardiac changes in sepsis at various stages, adjusting and guiding treatment in a timely manner, and optimizing hemodynamics.

The study will obtained the systolic and diastolic function indices of the left and right hearts through ultrasound, filling the gap of cardiac ultrasound indices for sepsis in China in the existing guidelines or expert consensus. Some studies have shown that EF <52% in men or EF <54% in women suggests abnormal left ventricular systolic function (8), and EF >70% suggests a hyperdynamic state of left ventricular function (9). Systolic function can also be assessed by obtaining the MAPSE using M-mode echocardiography. There are no consensus recommendations for abnormal MAPSE values; however, MAPSE <1 cm can indicate abnormal left ventricular systolic function (10). Diastolic function is a major determinant of left ventricular compliance, and diastolic dysfunction is common in septic myocardial suppression and a major predictor of mortality in patients with sepsis and septic shock (11). This can lead to a further increase in left ventricular end-diastolic pressure (LVEDP) by increasing left ventricular end-diastolic volume (12), subsequently increasing the pressure in the pulmonary, right heart, and body circulations, leading to increased extravascular lung water and tissue edema. The preferred method to evaluate diastolic function is the early diastolic mitral annular velocity (*e*') measured by TDI; the lower the value, the worse the diastolic function. The ratio of early diastolic transdiastolic inflow velocity (*E*) to early diastolic mitral annular velocity (*E*/*e*') correlates with left heart pressure and better reflects the increase in pressure (13). *e*' (at the septum) <7 cm/s or *e*' (at the lateral wall of the ventricle) <10 cm/s suggests abnormal left ventricular diastolic function, *E*/*e*' >14 cm/s suggests abnormal left ventricular diastolic function, and *E*/*e*' <8 cm/s suggests normal left ventricular diastolic function (14). The occurrence of acute respiratory distress syndrome and mechanical ventilation in patients with sepsis impacts right ventricular function, as right ventricular function is associated with afterload, which increases with hypoxemia, hypercapnia, elevated inspiratory pressure, and elevated positive end-expiratory pressure (15). The TAPSE is the simplest and most reproducible measure of right ventricular function, and a TAPSE <17 mm suggests abnormal right ventricular systolic function (16). A decrease in TAPSE is associated with increased mortality in critical illness (17).

In addition, septic cardiac changes exist in both directions, and there are currently no studies that consider both changes together in the same study. A previous radionuclide angiography study showed a reduced LVEF in a subgroup of patients with sepsis because the left ventricle, which undergoes dilatation in sepsis, can maintain beat volume if fluid resuscitation is adequate (18); however, more surprisingly, a study showed that patients with a reversible decrease in EF have a better prognosis than those without a decrease in EF (19), probably because septic myocardial suppression is a protective event that prevents the activation of cell death pathways by reducing energy expenditure in the presence of limited energy production and may allow the potential for full recovery of cellular function to be realized (20). Since the occurrence of “inhibition” may instead be beneficial, in line with the findings that left ventricular function in a hyperdynamic state affects mortality, it contradicts our previous knowledge that the occurrence of septic myocardial inhibition increases mortality in patients with sepsis (2). The prognostic differences between patients with normal, suppressed, and hyperdynamic cardiac phenotypes are unclear.

Finally, owing to the temporal variability in the onset of septic cardiac changes, this study was designed to collect data at four specific time points. One of the main clinical features of septic myocardial suppression is its apparent reversibility, with several studies reporting that patients can fully recover their cardiac function to the premorbid state (18, 21, 22). The changes detected by cardiac magnetic resonance imaging suggest myocardial edema or altered metabolic status, unlike the pattern of ischemia and necrosis, which are consistent with the characteristics of reversibility (23). This “reversibility” also suggests that there may be interconversion between the three states of cardiac suppression, hyperdynamic, and normal in sepsis patients, but the ratio and time of conversion are not clear, which is also important for triggering the timing of treatment initiation and withdrawal. Therefore, the type of septic cardiac changes at occurrence, timing of occurrence, duration, and prognostic impact of subsequent phenotypic alterations are also aims and innovations of this study.

Clinically, septic cardiac changes manifest as symptoms of circulatory failure associated with systemic infections. The differences in the clinical manifestations of cardiac insufficiency in patients with nonseptic decompensated heart failure lie in the alterations of overall hemodynamic parameters (preload, afterload, microcirculation). Unlike other cardiac diseases, cardiac changes in patients with sepsis require a multimodal approach for diagnosis and treatment. Unfortunately, there are no treatment recommendations specifically for the cardiac changes associated with sepsis. Patients with impaired left ventricular diastolic function may be at greater risk of fluid over-resuscitation, whereas patients with impaired left heart systolic function require more cardiac stimulant support, as volume resuscitation alone to correct perfusion deficits may be difficult to achieve; patients with right heart insufficiency require intensive treatment for acute respiratory distress syndrome and adjustment of mechanical ventilation parameters. In contrast, patients in a hyperdynamic left ventricular state require vasoconstrictors to improve vascular paralysis or symptomatic use of cardiac depressants.

Elevated plasma levels of troponin I and troponin T in patients with sepsis have been associated with LVSD and myocardial injury (24), as well as mortality (25). The mechanism of troponin elevation in sepsis may be caused by inflammation-induced cytoplasmic leakage from cardiomyocytes rather than cell death, and myocardial ischemia does not appear to be the culprit (26). BNP and NT-proBNP are hormones secreted by the myocardium in response to pressure stretching of the ventricular wall and have been proposed as markers of fluid loading status and early indicators of myocardial depression (27). However, other organ dysfunctions may affect troponin and BNP metabolism without being fully influenced by the actual myocardial injury and volume profile. In another study on coronary arteries in patients with sepsis, blood samples from the coronary sinus showed no increase in lactate production by cardiomyocytes, excluding myocardial ischemia as a cause of left ventricular dysfunction (28). Coronary angiography or CT imaging cannot be routinely performed in patients with sepsis with elevated troponin levels. Therefore, sepsis-induced cardiac changes should be defined as functional phenomena rather than as biochemical elevations. Echocardiography, which is widely used in clinical practice, has the advantage of being noninvasive and reproducible, making it the best way to assess cardiac changes in patients with sepsis.

The results of this study will enable medical students to understand the important value of ultrasound as a means of non-invasive hemodynamic monitoring in screening for etiology and dynamic assessment of disease in critically ill patients. In addition, this study was conducted under the guidance of the China Critical Ultrasound Study Group (CCUSG), and its research results are expected to become the organization's ultrasound training content to be applied and promoted in China and even around the world, which will help promote ultrasound in the promotion and application of residency and specialist physician training to improve the quality of medical teaching and residency training.

In conclusion, this study aimed to obtain data on patients with sepsis from multiple centers across the country, analyze each echocardiographic phenotype of sepsis, and investigate its incidence, duration, and prognosis. The prognosis of various patients can be compared as a predictor of future clinical prognosis, especially in those with “suppressed” and “hyperdynamic” phenotypes. This will be a novel predictor of clinical outcomes in patients with sepsis and the establishment of cardiac ultrasound phenotypes can also serve to guide and educate young physicians.

A limitation of this study may be that patients for whom transthoracic echocardiography data were not available did not undergo further transesophageal ultrasound due to medical constraints at each center and to avoid additional risks and lack of benefit to the patient. It is promising to study subgroups of sepsis patients with other underlying diseases, such as chronic hepatic and renal diseases, autoimmune diseases, and immunosuppressive states, as their underlying diseases may affect the prognosis by influencing the infection status. More specific and sensitive biomarkers for cardiac alterations in sepsis will also be an important area of future research and should be further explored in other studies.

## Ethics statement

The studies involving human participants were reviewed and approved by Ethics Committee of Fujian Provincial Hospital. The patients/participants provided their written informed consent to participate in this study.

## Author contributions

HongxZ: conceptualization, methodology, data curation, and writing—original draft. XW: validation and writing—review and editing. WY, HongmZ, LL, and PP: validation and data curation. YZ, WH, ZX, BY, CW, and TL: data curation. RY and XS: validation, writing—review and editing, and supervision. All authors contributed to the article and approved the submitted version.

## Funding

This protocol was financially supported by the High-level hospital foster grants from Fujian Provincial Hospital, Fujian Province, China [Grant number: (2020) HSJJ14].

## Conflict of interest

The authors declare that the research was conducted in the absence of any commercial or financial relationships that could be construed as a potential conflict of interest.

## Publisher's note

All claims expressed in this article are solely those of the authors and do not necessarily represent those of their affiliated organizations, or those of the publisher, the editors and the reviewers. Any product that may be evaluated in this article, or claim that may be made by its manufacturer, is not guaranteed or endorsed by the publisher.
